# Evaluation of a standardised protocol to measure the disease burden of respiratory syncytial virus infection in young children in primary care

**DOI:** 10.1186/s12879-021-06397-w

**Published:** 2021-07-26

**Authors:** J. J. G. T. van Summeren, C. Rizzo, M. Hooiveld, J. C. Korevaar, J. M. T. Hendriksen, M. L. A. Dückers, D. Loconsole, M. Chironna, M. Bangert, C. Demont, A. Meijer, S. Caini, E. Pandolfi, J. Paget

**Affiliations:** 1 Nivel, Netherlands Institute for Health Services Research, P.O. Box 1568, 3500BN Utrecht Utrecht, The Netherlands; 2 IRCCS, Bambino Gesù Children’s Hospital, Rome, Italy; 3 University of Groningen, Faculty of Behavioural and Social Sciences, Groningen, The Netherlands; 4 Department of Biomedical Sciences and Human Oncology-Hygiene Section, University of Bari, “Aldo Moro”, Bari, Italy; 5 Sanofi Pasteur, Lyon, France; 6 Centre for Infectious Diseases Research, Diagnostics and laboratory Surveillance, National Institute for Public Health and the Environment (RIVM), Bilthoven, The Netherlands

**Keywords:** Respiratory syncytial virus, Health care utilization, Socio-economic impact, General practice, Paediatrician, Infant, Child preschool, Quality of life

## Abstract

**Background:**

A better understanding of the burden of respiratory syncytial virus (RSV) infections in primary care is needed for policymakers to make informed decisions regarding new preventive measures and treatments. The aim of this study was to develop and evaluate a protocol for the standardised measurement of the disease burden of RSV infection in primary care in children aged < 5 years.

**Methods:**

The standardised protocol was evaluated in Italy and the Netherlands during the 2019/20 winter. Children aged < 5 years who consulted their primary care physician, met the WHO acute respiratory infections (ARI) case definition, and had a laboratory confirmed positive test for RSV (RT-PCR) were included. RSV symptoms were collected at the time of swabbing. Health care use, duration of symptoms and socio-economic impact was measured 14 days after swabbing. Health related Quality of life (HRQoL) was measured using the parent-proxy report of the PedsQL™4.0 generic core scales (2–4 years) and PedsQL™4.0 infant scales (0–2 years) 30 days after swabbing. The standardised protocol was evaluated in terms of the feasibility of patient recruitment, data collection procedures and whether parents understood the questions.

**Results:**

Children were recruited via a network of paediatricians in Italy and a sentinel influenza surveillance network of general practitioners in the Netherlands. In Italy and the Netherlands, 293 and 152 children were swabbed respectively, 119 and 32 tested RSV positive; for 119 and 12 children the Day-14 questionnaire was completed and for 116 and 11 the Day-30 questionnaire. In Italy, 33% of the children had persistent symptoms after 14 days and in the Netherlands this figure was 67%. Parents had no problems completing questions concerning health care use, duration of symptoms and socio-economic impact, however, they had some difficulties scoring the HRQoL of their young children.

**Conclusion:**

RSV symptoms are common after 14 days, and therefore, measuring disease burden outcomes like health care use, duration of symptoms, and socio-economic impact is also recommended at Day-30. The standardised protocol is suitable to measure the clinical and socio-economic disease burden of RSV in young children in primary care.

## Introduction

Respiratory syncytial virus (RSV) is the most common pathogen causing respiratory diseases in young children [1–4]. RSV can present in the form of a variety of clinical syndromes, including upper respiratory tract infections, or lower respiratory diseases such as bronchiolitis and pneumonia. RSV is highly seasonal and occurs mostly during winter season in temperate climates [5]. Sixty to 70 % of all children experience an RSV infection before the age of one, and nearly all do so before the age of two [6].

A global burden of disease study estimated that in 2015 approximately 33.1 million young children were infected with RSV, resulting in 3.2 million hospitalisations and 59,600 in-hospital deaths [1]. In Western countries, mortality due to an RSV infection is rare, however, annual hospitalisation rates in the first year of life are estimated to be 3.2–42.7 cases per 1000 children, with a hospital stay length ranging between two to 11 days, and 2–12% of cases requiring an intensive care unit admission [2, 7].

The burden of RSV in young children emphasizes the importance of efforts to develop new RSV interventions, for example non-pharmaceutical prevention strategies, immunization strategies or treatments. Current treatment options are limited to supportive care [8, 9], and the only available antiviral monoclonal antibody (mAb) ‘Palivizumab’ is considered cost-effective for certain high risk group infants and requires monthly injections during winter [10]. New candidate RSV vaccines and mAbs (with longer half-life times) are in late-stage clinical trials [11–13]. Therefore, accurate estimates of the burden of RSV, including in primary care, are crucial to better assess the overall impact RSV may have on the society.

‘Burden of disease’ is a general term without a universally accepted definition and refers to the human and economic costs that result from poor health. RSV ‘burden of disease’ studies in young children (aged 0–4 years), have mostly been focused on the morbidity and mortality rates of RSV infections [1, 6, 14, 15]. The socio-economic burden of RSV infections in young children has been studied, however, a meta-analysis showed that of the 365,828 RSV disease episodes included in cost-analysis studies, only 27,286 (7.4%) focused on outpatient and emergency cases [16]. To our knowledge, only two outpatient studies have prospectively investigated the clinical and socio-economic burden of laboratory confirmed RSV infections in young children; and both studies collected data in the early 2000s [17, 18]. More recently, one study has investigated the health care use, duration of illness and complications associated with RSV in a cohort of newborn infants [19]. There is therefore a lack of knowledge on the clinical and socio-economic disease burden of RSV infections in young children in primary care.

The aim of this study was to develop a protocol for the standardised measurement of the clinical and socio-economic disease burden of laboratory confirmed RSV infections in primary care in young children (< 5 years). Paediatric primary care in Europe has been categorised into three distinct groups: (1) paediatrician-led, (2) general practitioner led and (3) a combination of the two [20]. In Italy there is a paediatrician-led system for young children and in the Netherlands a general practitioner-led system. Therefore, the disease burden protocol was evaluated in Italy and the Netherlands. Considering routine influenza surveillance networks are implemented worldwide, the second aim was to evaluate whether it is possible to implement the disease burden protocol in an existing influenza surveillance network in order to better understand its potential for patient recruitment. In the Netherlands the protocol was therefore implemented in the existing influenza surveillance network.

## Methods

### Study design & setting

The RSV ComNet study was set up as a prospective cohort study in primary care with a follow up of 30 days. For each child (< 5 years) included in the study, data collection was performed at three moments in time, namely at the day of swabbing (Day 1), and after approximately 14 and 30 days. The disease burden protocol and the procedures for patient recruitment and data collection are described after the data analyses paragraph. To evaluate the disease burden protocol, we aimed to collect 400 swabs in Italy and 200 swabs in the Netherlands.

### Data analyses

For the evaluation of the disease burden protocol we used descriptive statistics to examine the feasibility of 1) patient recruitment, i.e. the weekly number of children swabbed, percentage of positive RSV cases, and the response rate on questionnaires, and 2) data collection procedures, i.e. the timing of questionnaires, duration time to complete the Day-14 and Day-30 questionnaires by parents, and the number of children recovered after 14 days. Moreover, descriptive statistics were used to present the baseline characteristics of the children included in the study. All analyses were conducted with Stata SE version 15.0 (StataCorp, 2013, College Station, TX).

In addition, we performed a qualitative process evaluation to assess the feasibility of the patient recruitment and data collection procedures using the experiences from the paediatricians, GPs, and the two research teams. In Italy the paediatricians were interviewed about their experiences through a structured questionnaire. In the Netherlands, GPs who attended the Annual Sentinel Network meeting were asked about their experiences. In addition, research nurses in Italy observed whether parents did understand the questions.

### Disease burden protocol

#### Eligibility criteria participants

Inclusion criteria were children, aged < 5 years, consulting a physician in primary care i.e. paediatrician or general practitioner (GP) with symptoms of an acute respiratory infection (ARI), and a laboratory confirmed diagnosis of RSV. The ARI case definition was based on the definition published by the World Health Organization (WHO) for community based surveillance [21, 22]. For this study we added the criteria: the physician judged that the illness is due to a respiratory infection. Exclusion criteria were insufficient knowledge of the national language by the parents, and insufficient intellectual abilities of the parents to complete the questionnaires.

#### Measurements and follow-up

**Fig. 1:**
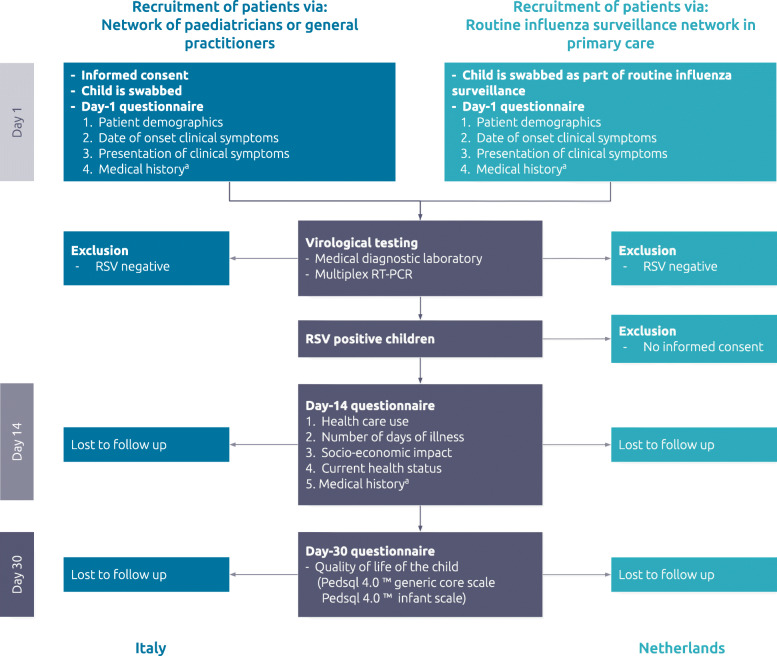
Flowchart of patient recruitment and data collection process. In Italy (dark blue boxes) patients were selected via a network of pediatricians involved in study, in the Netherlands (light blue boxes) patients were selected via the routine influenza surveillance system. In both countries, parents of RSV positive children were invited to complete the Day-14 and Day-30 questionnaires (grey boxes). In patients selected via the routine influenza surveillance (Netherlands), informed consent for swab collection was obtained via the routine surveillance procedures. In addition, parents of RSV positive children were asked for informed consent to participate in the study. ^a^The questions related to medical history were added to the Day-14 questionnaire in the Netherlands because it was not possible to complete the questions in the Day-1 questionnaire for practical reasons

At day 1 a nasopharyngeal swab or a nasopharyngeal and oropharyngeal swab was taken, and the physician completed a short questionnaire (Day-1) for each child (Fig. 1). The questionnaire included information about patient demographics, date of onset of symptoms, presenting symptoms and some relevant medical history of the child, and was based on a shortened version of the WHO surveillance form [23].

The swabs collected at day 1 were tested in medical diagnostic laboratories using multiplex RT-PCR. In addition, data regarding the weekly number of swabs, percentage of RSV positive cases, number of coinfections and RSV subtype were collected.

After approximately 14 days, the first parental questionnaire was completed regarding health care use of the child for the RSV infection, days of illness, socio-economic impact and current health status. The questionnaire was based on a previous study which investigated the disease burden of influenza and was adapted for the purposes of our population by the research team [24].

After approximately 30 days, the second parental questionnaire was completed. Quality of life was measured using the Paediatric Quality of Life Inventory (PedsQL)™ 4.0 generic core scales and PedsQL™ 4.0 infant scales. The parent-proxy report of the PedsQL™ 4.0 generic core scales is developed for children aged 2–18 years. The parent-proxy report of the PedsQL™ 4.0 infant scales has different versions for the age categories 1–12 and 13–24 months [25]. All PedsQL™ questionnaires were linguistically validated for the Italian and Dutch language. The PedsQL™ 4.0 generic core scales has shown good internal consistency and reliability, the initial measurement properties for the PedsQL™ 4.0 infant scales demonstrates that these versions may be utilized to measure the generic HRQoL [26–28] Questions regarding health care use and socio-economic impact were repeated when the child was still hospitalized after 14 days.

### Patient recruitment and data collection procedures

#### Italy

In Italy, children were recruited for the study and asked for informed consent by the paediatricians during the consultation. A network of paediatricians was established in two Italian municipalities: Rome (Lazio region) and Bari (Puglia region). The paediatricians (12 in each region) swabbed all children meeting the WHO ARI case definition and completed the Day-1 questionnaire. Swabs were sent by courier in the Lazio region and by parents of children swabbed in the Puglia region to the medical diagnostic laboratories (OBPG hospital or University of Bari). Specimens were analysed using a commercial kit multiplex RT-PCR (Allplex™ Respiratory Full Panel Assay) for 16 viruses (including Adenovirus, Influenza A, Influenza B, Parainfluenza 1, Parainfluenza 2, Parainfluenza 3, Parainfluenza 4, Respiratory syncytial virus A, Respiratory syncytial virus B, Metapneumovirus, Coronavirus OC43, Coronavirus 229E, Coronavirus NL63, Rhinovirus, Bocavirus, Enterovirus). All parents of children who tested RSV positive were contacted by telephone by research nurses to complete the follow-up questionnaires. Parents signed an informed consent form before swab collection. The medical ethical committee of OPBG Medical Centre Italy provided a waiver for ethical approval (Prot. N 1301), all methods were performed in accordance with the guidelines for good clinical practice.

#### The Netherlands

In the Netherlands, the infrastructure of the national routine influenza surveillance network in primary care was used [29]. For the routine surveillance program, 36 GP practices including 55 fulltime equivalent GPs, are asked to swab each week at least one child under the age of 10 years with influenza-like illness (ILI) or another ARI and complete the Day-1 questionnaire. Swabs were sent to RIVM by post and analysed using In-House RT-PCR for 6 viruses (Influenza A, Influenza B, RSV A, RSV B, Rhinovirus, Enterovirus). Among the children participating in the surveillance program, only parents of children under the age of 5 years with a positive RSV test result were asked for informed consent for this study via a letter sent by their GP. The follow-up questionnaires were collected using a digital questionnaire system. As the Day-1 questionnaire was collected as part of the influenza surveillance program in the Netherlands, the questions regarding the medical history of the child were added to the Day-14 questionnaire for practical reasons. The medical ethical committee of VU medical center provided a waiver for ethical approval, all methods were performed in accordance with the guidelines for good clinical practice.

## Results

### Feasibility of patient recruitment and data collection procedures

Children were recruited between weeks 45/2019 and 12/2020 in Italy and week 40/2019 and 14/2020 in the Netherlands. Patient recruitment ended prematurely in both countries due to the COVID-19 pandemic (lockdown measures). In Italy, 293 children were swabbed after parents had given informed consent for the study, 119 children (41%) were RSV positive and included in the study (Table 1). In the Netherlands, 152 children were swabbed in the influenza surveillance program, 32 children (21%) were RSV positive, the parents of 13 children (41%) gave informed consent for this study and 12 parents (38%) completed the Day-14 questionnaire. It is unknown whether the 19 parents that did not provide informed consent for this study were non-responders or were not willing to participate in this study.

**Table 1:** Indicators of the feasibility of patient recruitment and data collection procedures

	Italy	Netherlands
**Feasibility patient recruitment**		
Number of children swabbed (n)	293	152
Number of RSV positive children (n,%)	119 (41%)	32 (21%)
Response rate Day-14 (n, %)	116 (98%)	12 (38%)
Response rate Day-30 (n, %)	116 (98%)	11 (34%)
**Feasibility data collection procedures**	N = 119	N = 12
Days between disease onset and swab (median, IQR)	2 (1–3.5)	3 (2–4.5)
Days between swab and Day-14 (median, IQR)	17 (14.5–20.5)	20 (16.5–30.5)
Days between swab and Day-30 (median, IQR)	32.5 (30.5–35)	36.5 (29.5–39)
Having symptoms at Day-14 (n, %)	40 (34%)	8 (67%)
Questionnaire duration, Day-14 (minutes)	7 (5–10)	4 (3–5)
Questionnaire duration, Day-30 (minutes)	10 (7–15)	4 (3–8)

Note. In Italy all parents gave informed consent before swabbing, while in the Netherlands parents were asked for informed consent after the child was tested RSV positive (Fig. 1). In the Netherlands, 19 parents of RSV positive children did not respond on the study letter asking for informed consent, and 1 parent provided informed consent but did not complete the Day-14 questionnaire. This is reflected in the response rate at Day-14

As planned, children included in the study had acute symptoms (Table 1). The first parental questionnaire (Day-14) was completed after a median of 17 days in Italy and 20 days in the Netherlands and the second parental questionnaire (Day-30) after 32.5 days and 36.5 days, respectively. On average parents needed between 4 and 10 min to complete one questionnaire (Day-14 or Day-30). In total, 48 parents (36%) reported the child was having symptoms after 14 days.

### Process evaluation of patient recruitment and data collection procedures

In Italy, it was feasible to implement the standardised protocol in the routine care provided by the network of paediatricians. It was planned that paediatricians in Rome arranged a courier for the transportation of the swabs to the reference laboratory multiple times a week. However, it was more feasible for paediatricians when this was organised by a member of the research team. Therefore, this procedure was adapted during the study. In Bari, the parents brought the swabs to the reference laboratory themselves and this procedure worked smoothly. The increase in workload for paediatricians and laboratories was experienced as minimal as they recognized the added value of having diagnostic test results at primary care level.

In the Netherlands, the research team indicated that it was feasible to implement the standardised protocol in the influenza surveillance network in primary care. The routine influenza surveillance network was economical (as it uses an existing infrastructure e.g. it uses existing logistical procedures for the transportation of swabs to the reference laboratory). However, one important disadvantage was that it was not possible to collect informed consent directly at the time of swabbing as this would disrupt the influenza surveillance system. Informed consent for this study was therefore collected via a letter sent to the parents of children testing RSV positive. As a result, only 12 of the 32 parents of the RSV positive children responded to the request for informed consent and signed the informed consent for this study and completed the Day-14 questionnaire, while in Italy all parents gave informed consent for this study before swabbing and the response rate on the Day-14 questionnaire was 98% (Table 1).

### Day-14 and Day-30 questionnaires

Research nurses observed no difficulties for parents to complete the questions related to health care use, duration of symptoms, socio-economic impact and clinical symptoms. More difficulties were observed for parents to answer the questions regarding the child’s HRQoL using the PedsQL 4.0™ generic core scales and the PedsQL 4.0™ infant scales. Questions related to physical symptoms were difficult for parents with children under the age of 12 months. Questions regarding physical functioning (for example related to low energy level, participation in active playing, and for children over 12 months also about walking and running) were difficult to answer for parents with children under the age of 2 years, because the children did not have all the skills asked for in the questionnaire. For example, walking independently is mostly achieved between 10 and 18 months of age. For parents with children in all age categories, it was difficult to answer questions about emotional (i.e. feeling afraid or scared, feeling sad or blue, feeling angry or worrying) and social functioning (i.e. playing with other children or other children do not want to play with their child). Especially for the parents of children that did not have walking or talking skills, it was difficult to answer the questions about emotional and social functioning.

### Baseline characteristics of children with RSV in primary care

Baseline characteristics of children included in the study are shown in Table 2. RSV was the most common in children under the age of 1 year. The majority of children (73%) consulting in primary care had lower respiratory tract symptoms and 120 children (92%) did not have any chronic comorbidities or were born premature. None of the children were hospitalized after 14 days. A more comprehensive analysis of the disease burden outcomes in Italy will be described in another publication.

**Table 2:** Baseline characteristics and virological test results of RSV positive children in Italy (*n* = 119) and the Netherlands (*n* = 12)

	Italy				Netherlands
	Total (*n* = 119)	1–12 months (*n* = 53)	13–24 months (*n* = 26)	2–4 years (*n* = 40)	Total (*n* = 12)
*Boys*	59 (50%)	30 (57%)	13 (50%)	16 (40%)	9 (75%)
*Age in months median (IQR)*	15 (7–30)	6 (4–9)	20 (15–21)	35 (30–46)	11 (8–15)
***Symptoms***					
Cough	117 (98%)	52 (98%)	26 (100%)	39 (98%)	10 (83%)
Coryza	106 (89%)	46 (87%)	26 (100%)	34 (85%)	8 (67%)
Shortness of breath	89 (76%)	45 (87%)	15 (60%)	29 (73%)	6 (50%)
Sore throat	36 (30%)	16 (30%)	10 (38%)	10 (25%)	0
***Medical history***^***a***^					
*Prematurity*	6 (5%)	0	4 (15%)	2 (5%)	1 (8%)
*Other chronic medical condition*	2 (2%)	0	0	2 (5%)	0
*Chronic respiratory disease*	1 (1%)	0	0	1 (3%)	0
*Malnutrition*	0	0	0	0	1 (8%)
***Virological test results***					
*RSV A*	91 (76%)	40 (75%)	21 (81%)	30 (75%)	9 (75%)
*RSV B*	28 (24%)	13 (25%)	5 (19%)	10 (25%)	3 (25%)

Note: ^a^None of the children were immunocompromised or diagnosed with a previous RSV infection this season

## Discussion

To our knowledge, this is the first study that has developed and evaluated a standardised protocol to measure the clinical and socio-economic burden of RSV infections in young children in primary care. The study demonstrates that the standardised protocol, with small adaptations (see discussion below), is suitable to collect data to measure the clinical and socio-economic burden of RSV infections in young children in primary care. The disease burden outcomes for the 119 children included in Italy will be described elsewhere. For the Netherlands, the data collection period is extended to recruit more children.

### Recommendations for the disease burden protocol

**Fig. 2:**
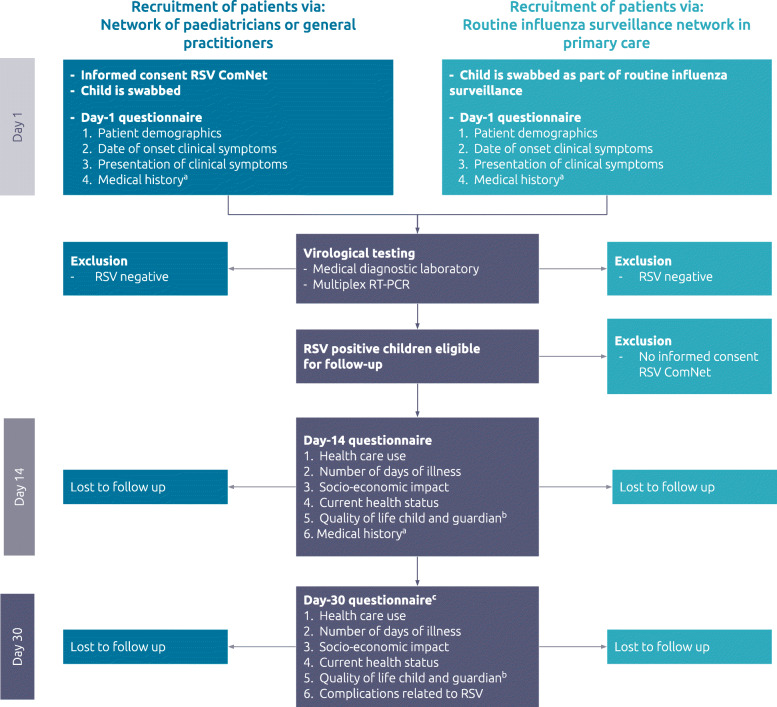
Final protocol to measure the clinical and socio-economic disease burden in primary care in young children. Children can be selected via a network of pediatricians or GPs involved in study (dark blue boxes), or via a routine influenza surveillance network in primary care (light blue boxes). Parents of RSV positive children will complete the Day-14 and Day-30 questionnaires (grey boxes). In children selected via the routine influenza surveillance program informed consent for swab collection is obtained via the routine surveillance procedures. Parents of RSV positive children are asked for informed consent for this study and will be asked to complete the Day-14 and Day-30 questionnaires. ^a^ Questions related to medical history are added to the Day-14 questionnaire in countries where it is not possible to complete the questions in the Day-1 questionnaire for practical reasons. ^b^ Quality of life is measured with one question on the child’s health status of today and one question on the guardian’s health status of today measured on a Visual Analogue Scale. ^c^ Topics 1 (health care use) and 3 (socio-economic impact) in the Day-30 questionnaire are introduced with a general question to examine whether the more detailed questions are required

Based on our results, we recommend that the following points in the original protocol are maintained: the overall study design, eligibility criteria of participants, and the timing of the measurements. Small changes in the data collected via the questionnaires are recommended. Figure 2 summarises the updated standardised protocol.

One of the changes that we recommend is that the questions related to health care use, duration of illness, and socio-economic impact are extended to the Day-30 questionnaire because RSV related symptoms were often reported by parents after 14 days. Secondly, complications associated with RSV, like acute otitis media and pneumonia are recommended to measure in the Day-30 questionnaire [19]. In addition, we recommend that more detailed information is collected on the clinical symptoms at baseline to examine whether children in primary care have lower or upper respiratory tract symptoms. Besides, information on the socio-economic impact of the child’s RSV infection on both parents is recommended.

Most importantly, our study showed that parents found it very difficult to assess the HRQoL of their young child. Not only were the questions difficult to answer for the parents, but the recall period of the HRQoL questionnaire was 30 days. This may be too long to measure HRQoL because of the RSV infection, given RSV is an acute short-term disease (57% of the parents reported the child had no symptoms after 14 days). Therefore, we think the PedsQL™ 4.0 generic core scales and PedsQL™ 4.0 infant scales were not an ideal tool to measure HRQoL in young children with an acute short-term disease like RSV. As information concerning QoL is very relevant for decision making regarding the introduction of new treatments or preventive measures, we recommend two general questions estimating the QoL of the child and the guardian are added to the questionnaire, the latter as a proxy of the impact on the family (Fig. 2).

To our knowledge, no previous studies have reported the clinical symptoms and medical history of young children with RSV in primary care. A previous study found that 21% of the hospitalized children had chronic comorbid conditions and 18% were born prematurely, while our study showed that 3% of the children in primary care had chronic comorbid conditions and 5% were born prematurely [30]. This would suggest that in contrast to hospitalized children the majority of children with RSV in primary care are otherwise healthy and, therefore, disease burden measurements in primary care are important.

### Recommendations for patient recruitment

The difference in the number of children included in Italy (*n* = 119) and the Netherlands (*n* = 12) was to a large extent expected, as the number of children eligible for recruitment was different between countries (see methods).

The disease burden protocol was developed to measure the burden of RSV on an individual patient level. However, if (1) a predetermined random selection of children with ARI symptoms are swabbed; (2) information on the enlisted population is available; and (3) the size of the study population is appropriate; it would be possible to calculate population-based estimates of the incidence of RSV in primary care. All these three conditions are met for the data collected in Italy. In that situation, the standardised protocol can also be used to estimate the socio-economic costs related to RSV infections in primary care on a regional or country level.

The patient recruitment strategy that best fits the data collection procedure can differ between countries. The process evaluation showed that it is feasible to recruit children via a network of paediatricians, and to select children via a routine influenza surveillance network in primary care. The advantage of recruiting children via a network of physicians (e.g. paediatricians in Italy) is that more easy a predetermined random selection of children with ARI symptoms can be recruited (for example all children), which is one of the requirements to make population-based estimates. On the other hand, it is more time-consuming and more expensive to arrange the logistical procedures. The advantage of recruiting via an existing influenza surveillance network is that all the logistical procedures already exist. A disadvantage is that a surveillance network has to be available, recruitment of children is based on the case definitions used in the surveillance system (in most countries children with ILI or ARI symptoms are eligible), and the recruitment of children under the age of 5 years is usually not the main focus of the surveillance program. Therefore, the disease burden is measured on an individual patient’s level, however, calculation of population-based estimates might be more challenging in this situation. Another point of attention is that the size of the surveillance system needs to be appropriate to include the desired number of children. Small adaptations to the influenza surveillance network might be helpful to increase the number of included children (e.g. increase swabbing in young children or increase the percentage of RSV positives by using the WHO ARI case definition for RSV in young children). Another option is that patient recruitment is planned for 2- or 3 seasons.

### Measurement of HRQol with the PedsQL™ 4.0 generic core scales and infant scales

Measuring HRQoL in very young children is known to be challenging. There are several parent-proxy reports for children older then 2 years of age, but only three instruments to measure HRQoL in infants under the age of 2 years [28, 31, 32]. The PedsQL™4.0 infant scales was, to our knowledge, the only questionnaire that has different versions for the age categories 1–12 months and 13–24 months [28]. The initial feasibility, internal consistency, reliability and validity of this questionnaire was established and showed that the questionnaire might be used to measure generic HRQoL in children under the age of 2 years [28]. However, the PedsQL™4.0 infant scales are not widely used yet. Our study showed that it was very difficult for parents to estimate the HRQoL of very young children.

## Conclusions

Our study showed that we have developed a standardised protocol that is appropriate to measure the clinical and socio-economic disease burden in young children in primary care. We are planning the implementation of the standardised protocol in other European countries as part of the RSV Community Network (RSV ComNet) [33]. The experiences gained from this evaluation study will allow us to provide guidance for the implementation of the protocol in these new countries, which is important as each country has their own health care system and respiratory surveillance infrastructure. The disease burden estimates examined in these studies will be important for policy makers to make informed decisions regarding new RSV interventions.

## Data Availability

The datasets used and/or analysed during the current study are available from the corresponding author on reasonable request.

## Abbreviations

RSV: Respiratory syncytial virus
RT-PCR: Reverse transcription polymerase chain reaction
PedsQL: Pediatric quality of life inventory
HRQoL: Health related Quality of life
GP: General practitioner
ARI: Acute respiratory infection
WHO: World Health Organization
ILI: Influenza-like illness
